# Effects of staining and artificial aging on optical properties of gingiva-colored resin-based restorative materials

**DOI:** 10.1007/s00784-022-04643-2

**Published:** 2022-07-26

**Authors:** Vesna Miletic, Branka Trifković, Dejan Stamenković, Rubens Nisie Tango, Rade Dušan Paravina

**Affiliations:** 1 Sydney Dental School, Faculty of Medicine and Health, The University of Sydney, Surry Hills, NSW 2010 Australia; 2 Clinic for Prosthodontics, School of Dental Medicine, University of Belgrade, Belgrade, Serbia; 3 Private Practice, Belgrade, Serbia and John M Powers, PhD, Houston Center for Biomaterials and Biomimetics (HCBB), University of Texas School of Dentistry at Houston, Houston, TX USA; 4 Department of Dental Materials and Prosthodontics, State University of Sao Paulo School of Dentistry at Sao Jose dos Campos, Sao Jose dos Campos, Brazil and, John M Powers, PhD, Houston Center for Biomaterials and Biomimetics (HCBB), University of Texas School of Dentistry at Houston, Houston, TX USA; 5 Department of Restorative Dentistry and Prosthodontics and John M Powers, PhD, Houston Center for Biomaterials and Biomimetics (HCBB), University of Texas School of Dentistry at Houston, Houston, TX USA

**Keywords:** Artificial aging, Color, Composites, Gingiva-colored, Gloss, Translucency

## Abstract

**Objectives:**

To evaluate CIEDE2000/CIELAB differences in color (Δ*E*_00_/Δ*E*_ab_), and translucency parameter (ΔTP_00_/ΔTP_ab_), and gloss of gingiva-colored resin-based restorative materials upon staining/aging.

**Materials and methods:**

Disc-shaped, 10 mm in diameter, and 2-mm-thick samples (*n* = 5/group) were made from giomer (Beautifil II gingiva), oligomer-based (crea.lign GUM gel), CAD/CAM polymethyl-methacrylate-based (IvoBase CAD), PMMA-based (ProBase Hot), and dimethacrylate-based (SR Nexco Paste Gingiva). Color and gloss were recording using a benchtop spectrophotometer and gloss meter, respectively, at baseline (T0), and upon staining in coffee or red wine for 60 (T1) and 120 h (T2), or artificial aging of 150 kJ/m^2^ (T1) and 300 kJ/m^2^ (T2). Three-way analysis of variance (materials x staining conditions x time intervals), Tukey’s test (*α* = 0.05), and Pearson’s correlation test were used in analytical statistics.

**Results:**

CIEDE2000 color differences ranged from 1.0 to 4.4 (coffee), 1.5 to 5.3 (wine), and 0.9 to 2.0 after artificial aging, with Δ*E*_00_ values being significantly higher for Beautifil than other materials (*p* < 0.05). ΔTP_00_ values ranged from 0.2 to 0.7 and were statistically higher upon staining in wine compared to artificial aging (*p* < 0.05). Gloss values at T0 were 76.7–87.0. Beautifil exhibited the lowest gloss retention (50.8–60.2%) after staining, compared to > 90% of other materials (*p* < 0.05). Δ*E*_00_/Δ*E*_ab_ and ΔTP_00_/ΔTP_ab_ were positively correlated (*p* < 0.0001).

**Conclusions:**

Color, translucency, and gloss changes of gingiva-colored restorative materials were material- and staining/aging-dependent. Generally, wine caused greatest changes in color (with IvoBase CAD being the most color stable) and translucency parameter. All materials except Beautifil gingiva II exhibited staining- and aging-dependent gloss retention greater than 90% for all compared time intervals.

**Clinical relevance:**

Optical properties of resin-based gingiva-colored restorative materials depend on material, staining/aging conditions, and exposure time. Certain materials should be avoided in individuals with high consumption of red wine and coffee.

## Introduction

Expansion of fixed prosthodontics, especially implant-supported dentures, has increased focus on artificial gingival tissue replacements. Gingiva-colored or gingiva-colored-colored resins, composites, and ceramics are used to restore gingival tissue from isolated gingival recession cases to large defects and bone resorption [1]. Gingival color is gaining attention from an esthetic point of view, in terms of tooth-gingiva harmony [2, 3], gingiva-colored material-natural gingiva match [4], color perception in the gingival chromatic space [4–6], and color stability of gingiva-colored resin-based materials [7–9].

Historically, color differences related to teeth and restorative materials were determined using the CIELAB formula (Δ*E*_ab_), based on three coordinates in the color space: L* indicating lightness (L* = 100 white, L* = 0 black), a* indicating green–red (− a* green, + a* red), and b* indicating blue-yellow (− b* blue, + b* yellow) color coordinate. However, the newer CIEDE2000 formula with corresponding color differences (Δ*E*_00_) is recommended as it outperforms CIELAB and better correlates with visual findings [10].

The true clinical relevance of comparison of color differences in dentistry lies in their relationship to perceptibility and acceptability thresholds (PT and AT, respectively), more so than in mere statistical significance. Color difference thresholds have been established in dentistry [11], including dental ceramics [12] and skin-colored elastomers [13]. Recently, color difference thresholds were reported using gingiva-colored porcelain and computer-based tooth-gingiva simulations [5, 14]. The reported PT and AT for gingival shades determined using CIELAB and CIEDE2000 formulas are not quite in unison [5, 6, 14]. The Δ*E*_00_ values for 50:50% PT and AT for “gingiva-colored” were found to be generally higher, 2.1 (PT) and 2.9 (AT) [5], than those for “white” esthetics, 0.8 (PT) and 1.8 (AT) [11]. Color differences for gingiva-colored materials are categorized as follows: excellent match, acceptable match, mismatch type [a], mismatch type [b], and mismatch type [c] [15].

Color stability is usually determined by immersion of samples in colored beverages, most often red wine and coffee, due to their strong staining potential. Unlike tooth-colored restorative materials [16–22], optical properties of gingiva-colored resin-based restorative materials have not been extensively studied. Effects of staining in various colored beverages on surface properties were evaluated for gingiva-colored composites [7, 8, 23] and CAD/CAM or resin-based denture base materials [24–26]. The most commonly used staining solutions were red wine and coffee [7, 8, 13, 24, 26], but tea, curry solution [8, 25], coca-cola [24, 26], and mouth rinse chlorhexidine [13] were also used to test color difference in gingiva-colored resin-based materials.

According to International Organization for Standardization (ISO) 4892–2 standard, color stability of restorative materials is evaluated following artificial accelerated aging using a xenon lamp in weathering and lightfastness test chamber [27]. This system consists of visible light, UV radiation, wet and dry conditions, and heating cycles. Studies using artificial aging on tooth-colored materials have reported higher Δ*E*_00_ for resin-based CAD/CAM [28] and laminate veneering materials [29] than their than ceramic counterparts for the same indications. A recent study on tooth-colored CAD/CAM materials with two aging cycles showed material-dependent Δ*E*_00_ which was not related to the presence of resin in the ceramic material [16]. Experimental resin mixtures containing different photoinitiators have shown lower Δ*E*_00_ for camphorquinone than type I phosphine oxide photoinitiators [30]. The effects of the artificial aging protocol on gloss, color, and translucency parameter (TP) of gingiva-colored restorative materials have not been evaluated.

The aim of this study was to determine and compare optical properties of gingiva-colored-colored, resin-based restorative materials following staining in red wine or coffee or accelerated artificial aging according to ISO 4892–2 standard. The research hypotheses were that (1) there would be statistically difference in color, translucency, and gloss among gingiva-colored-colored materials, based on (a) material, (b) staining (red wine, coffee)/aging, and (c) the exposure interval; and (2) there would be positive correlation between CIEDE2000 and CIELAB differences in color and translucency.

## Materials and methods

This experimental in vitro research study included five gingiva-colored resin-based materials which were used in the study (Table 1). Standardized, disc-shaped samples, 2 mm thick and 10 mm in diameter (*n* = 15), were prepared according to manufacturers’ instructions. Beautifil II gingiva, crea.lign GUM, and SR Nexco Paste Gingiva were light-cured with high-intensity LED light-curing unit (Elipar S, 3 M ESPE, St. Paul, MN), operating at ~ 1470mW/cm^2^. ProBase Hot was mixed in the dose of 1 graduation mark powder and 10 mL monomer and left in a closed cup for 10 min. Prepared samples were boiled in water at 100 °C for 45 min.

**Table 1:** Study materials and respective acronyms, manufacturers, composition, shade and batch numbers

Material	Manufacturer	Composition	Polymerization	Shade	LOT #
Beautifil II gingiva (Beautifil)	Shofu, San Marcos, CA	BisGMA, TEGDMA, S-PRG filler, initiator, pigments, others	Light-curing	G—light gingiva-colored	81705
crea.lign GUM (crea.lign)	Bredent, Senden, Germany	High-strength oligomer matrix, nano-ceramic filler (50%)	Light-curing	G2 rosa	N190304
IvoBase CAD (IvoBase)	Ivoclar Vivadent, Schaan, Liechtenstein	PMMA (> 90%), co-polymer, pigments	Industrially manufactured discs	Gingiva-colored	Y04913
ProBase Hot (ProBase)		Powder: PMMA (> 95%), softening agent, benzoyl peroxide (< 1.5%), pigments Liquid: MMA, DMA, catalyst	Heat-curing	-	XT0184
SR Nexco Paste Gingiva (Nexco)		DMA (17–19 wt.%); copolymer and silicon dioxide (82–83 wt.%), stabilizers, catalysts, pigments, 10–100-nm inorganic fillers (64–65 wt.%)	Light-curing	G2	XZ1105

*BisGMA*, bisphenol A glycidil methacrylate; *TEGDMA*, triethylene glycol dimethacrylate; *S-PRG*, fluoroboroaluminosilicate glass; *PMMA*, polymethyl methacrylate; *MMA*, methyl methacrylate; *DMA*, dimethacrylate

Power and sample size analysis was done in G*Power v3.1 (www.psychologie.hhu.de) for “Means – Many groups ANOVA: main effects and interactions.” The required sample size per group to detect a large effect size *f* = 0.04 with alpha 0.05, power 0.95, numerator df 16 (5 materials, 3 staining conditions, and 3 intervals), and number of groups 45 resulted in 4.3 specimens per group. Therefore, it was decided to use 5 specimens per group.

Samples were polished under water cooling using # 600 grit SiC paper per 15 s in a grinding machine (Ecomet 6, Buehler, Lake Bluff, IL) at 150 rpm under mild hand pressure, after which were polished with Enhance PoGo Discs (Dentsply Sirona, Charlotte, NC) mounted in a low-speed handpiece (maximum 15,000 rpm) using mild hand pressure for 40 s. The same operator (R.N.T.) performed polishing of all samples. Samples were cleaned with deionized water for 10 min in an ultrasonic cleaner (Branson Ultrasonics, Brookfield, CT) and air-dried for 20 s for baseline (T0) color, TP, and gloss measurements, after which they were divided into 3 subgroups (*n* = 5), exposed to coffee or red wine staining or accelerated artificial aging.

Samples were stored in staining solution in an incubator at 37 °C in the dark for 60 (T1) and 120 h (T2), with solutions being changed once a day. Cabernet Sauvignon red wine (Frontera, Concha y Toro, Santiago, Chile) was used for red wine staining. Filter coffee was prepared by mixing 2 tablespoons of ground coffee (Folgers Classic Medium Roast ground coffee, Folger Coffee, San Francisco, CA) to 200 mL of water. Specimens were rinsed with water after staining to remove excess wine/coffee and blot-dried with paper towels prior to repeated measurements. Artificial accelerated aging was performed according to International Organization for Standardization (ISO) 4892–2 standard, using a xenon lamp weathering and lightfastness test chamber (Suntest XXL + , Ametek Atlas, Mt. Prospect, IL) [27]. The artificial aging cycle consisted of light exposure (102 min) and water spraying (18 min) under artificial daylight (CIE D65 illuminant) at constant temperature (37 °C ± 3 °C) and relative humidity (50% ± 10%), with a black panel temperature of 65 °C and irradiance control in the 300- to 400-nm interval of 60 W/m^2^. The total energy delivered for artificial accelerated aging was 150 kJ/m^2^ (T1) and 300 kJ/m^2^ (T2), respectively.

Color and TP measurements were performed using a benchtop spectrophotometer Ci7600 (X-Rite, Grand Rapids, MI). Reflection values were recorded at 10-nm increments, using the following setup: CIE D65 standard illuminant, 2 degrees 1931 standard observer, specular component included, UV component included, small area view aperture (SAV, 6 mm in diameter). Prior to measurements, the spectrophotometer was calibrated per the manufacturer’s instructions. Color measurements were performed against white and black calibration tiles and provided comparisons within the same phase of the experiment. Reflection values were converted into CIELAB color differences (Δ*E*_ab_) using the following formula [31]:

$${\Delta E}_{\mathrm{ab}}={\left({{\Delta L}^{*}}^{2}+ {{\Delta a}^{*}}^{2}+ {{\Delta b}^{*}}^{2}\right)}^\frac{1}{2},$$

where L*, a*, and b* denote lightness, green–red, and blue-yellow coordinates, respectively, while Δ*E*_ab_ denotes CIELAB color difference. The CIELAB values were converted into CIEDE2000 *L*′, *C*′ (chroma), and *h*′ (hue) values, while Δ*E*_00_ color differences were calculated using the following formula [10]:

$${\Delta E}_{00}={\left[{\left(\frac{\Delta {L}^{^{\prime}}}{{K}_{\mathrm{L} }{S}_{\mathrm{L}}}\right)}^{2}+ {\left(\frac{\Delta {C}^{^{\prime}}}{{K}_{\mathrm{C} }{S}_{\mathrm{C}}}\right)}^{2}+ {\left(\frac{\Delta {H}^{^{\prime}}}{{K}_{\mathrm{H} }{S}_{\mathrm{H}}}\right)}^{2}+{R}_{T}\left(\frac{\Delta {C}^{^{\prime}}}{{K}_{\mathrm{C}}{S}_{\mathrm{C}}}\right)\left(\frac{{\Delta H}^{^{\prime}}}{{K}_{\mathrm{H}}{S}_{\mathrm{H}}}\right)\right]}^\frac{1}{2},$$

where Δ*L*′, Δ*C*′, and Δ*h*′ are differences between the corresponding color coordinates, computed based on the uniform color space used in CIEDE2000 and where *K*_L_*S*_L_, *K*_C_*S*_C_, and *K*_h_*S*_h_ are empirical terms for converting the differences for each coordinate into the CIEDE2000 difference formula.

CIELAB and CIEDE 2000 TP_ab_ and TP_00_ values were calculated utilizing the following formula [31, 32]:

$${\mathrm{TP}}_{\mathrm{ab}}={\left[{\left({L}_{\mathrm{W}}-{L}_{\mathrm{B}} \right)}^{2}+ {\left({a}_{\mathrm{W}}-{a}_{\mathrm{B}} \right)}^{2}{+ \left({b}_{\mathrm{W}}-{b}_{\mathrm{B}} \right)}^{2}\right]}^\frac{1}{2}$$

where *L*, *a*, and *b* denote lightness, green–red, and blue-yellow coordinates, respectively, against white (_W_) and black (_B_) backgrounds, and

$${\mathrm{TP}}_{00}= {\left[{\left(\frac{{{L}^{^{\prime}}}_{\mathrm{B} }- {{L}^{^{\prime}}}_{\mathrm{W}}}{{K}_{\mathrm{L}}{S}_{\mathrm{L}}}\right)}^{2 }+{\left(\frac{{{C}^{^{\prime}}}_{\mathrm{B}}- {{C}^{^{\prime}}}_{\mathrm{W}}}{{K}_{\mathrm{C}}{S}_{\mathrm{C}}}\right)}^{2 }+ {\left(\frac{{{H}^{^{\prime}}}_{\mathrm{B}}- {{H}^{^{\prime}}}_{\mathrm{W}}}{{K}_{\mathrm{H}}{S}_{\mathrm{H}}}\right)}^{2 }+\mathrm{RT} {\left(\frac{{{C}^{^{\prime}}}_{\mathrm{B} }- {{C}^{^{\prime}}}_{\mathrm{W}}}{{K}_{\mathrm{C}}{S}_{\mathrm{C}}}\right)}\left(\frac{{{H}^{^{\prime}}}_{\mathrm{B}}- {{H}^{^{\prime}}}_{\mathrm{W}}}{{K}_{\mathrm{H}}{S}_{\mathrm{H}}}\right)\right]}^{1/2}$$

where *L*, *C*, and *H* denotes lightness, chroma, and hue, respectively, against white (*_W_) and black (*_B_) backgrounds. RT is the rotation function that accounts for the interaction between hue and chroma differences in the blue region. *S*_L_, *S*_C_, and *S*_H_ adjust the total color difference for variation in the location of the color difference sample over the B and W backgrounds in *L*′, *a*′, and *b*′ coordinates and the parametric factors, *K*_L_, *K*_C_, *K*_H_, are correction terms for experimental conditions.

Gloss Meter (Novo-Curve, Rhopoint Instruments, St. Leonards-on-Sea, UK) was used for gloss measurements (GU, gloss units). Gloss was measured at T0, T1, and T2 periods, and gloss retention (GR, %) was expressed as percentage of GU at T0, at T1, at T2, and between T1 and T2.

Three measurements were performed per specimen at each time interval and the mean value was used for statistical analysis. Data for baseline (T0) Δ*E*_00_, Δ*E*_ab,_ ΔTP_00_, ΔTP_ab_, gloss (GU), and gloss retention (GR, %) for T0–T1, T0–T2, and T1–T2 were first checked for normal distribution using the Shapiro–Wilk test as a precondition for parametric testing. Data were then submitted to three-way analysis of variance (materials x staining conditions x time intervals) and Tukey post hoc test for inter-group comparison, both with *α* = 0.05 (Minitab 16, Minitab LLC, State College, USA). Pearson’s correlation coefficient was used to correlate Δ*E*_00_ with Δ*E*_ab_ and TP_00_ with TP_ab_ at different time intervals. Regression equation was used to calculate Δ*E*_00_ from known Δ*E*_ab_ and vice versa.

## Results

Means (SD) and analytical statistics for staining and aging-dependent CIEDE2000 color differences of evaluated materials and time intervals are presented in Table 2. Beautifil showed significantly higher Δ*E*_00_ than other tested materials after staining in both coffee and wine at T0–T2. At T0–T1, Beautifil had significantly higher Δ*E*_00_ values than other materials after coffee staining while the same was true for T1–T2 after wine staining (*p* < 0.05). Corresponding mean Δ*E*_ab_ values at the final observation period (T0–T2) were in the range of 2.1–6.1 for Beautifil, 1.0–2.9 for IvoBase CAD, 1.3–2.4 for crea.lign, 1.0–2.4 for Probase, and 1.0–2.9 for Nexco. In most cases, differences in Δ*E*_00_ and Δ*E*_ab_ did not reach statistical significance between observation intervals, except for Beautifil after wine/coffee staining (T0–T1: Δ*E*_00_ = 3.0; T0–T2: Δ*E*_00_ range 4.4–5.3; T1–T2: Δ*E*_00_ range 1.6–2.5) and Nexco after coffee staining which exhibited statistically greater Δ*E*_00_ at T0–T2 (mean Δ*E*_00_ = 1.3) compared to T0–T1 (mean Δ*E*_00_ = 0.9). Comparing T0–T1 and T1–T2 observation times, higher mean Δ*E*_00_ and Δ*E*_ab_ occurred in the first interval T0–T1 but failed to reach statistical significance except in the case of Beautifil which showed significantly higher Δ*E*_ab_ at T0–T1 after staining in wine (*p* < 0.05). Wine resulted in greater Δ*E*_00_ and Δ*E*_ab_ followed by coffee and artificial aging in all materials, except IvoBase CAD which showed greatest Δ*E*_00_ and Δ*E*_ab_ after artificial aging. The *R*^2^ values for the three-way ANOVA for Δ*E*_00_ and Δ*E*_ab_ were 78.4% and 78.8%, respectively.

**Table 2:** Means (SD) for staining- and aging-dependent changes in Δ*E*_00_ of evaluated materials and time intervals (staining: T0—baseline, T1 and T2—60-h and 120-h exposure, respectively, and artificial aging T0—baseline, T1 and T2—150 kJ/m^2^ (T1) and 300 kJ/m^2^ (T2), respectively)

Δ*E*_00_	Material	Staining/aging	T0–T1	T0–T2	T1–T2
Δ*E*_00_	Beautifil	C	3.0 (0.3)^AB,a^	4.4 (0.6)^A,a^	1.6 (0.4)^B,ab^
		W	3.0 (1.2)^B,ab^	5.3 (2.3)^A,a^	2.5 (1.7)^B,a^
		A	1.3 (0.2)^A,b^	1.7 (0.1)^A,b^	0.4 (0.1)^A,b^
	IvoBase	C	0.5 (0.1)^A,b^	1.0 (0.1)^A,b^	0.4 (0.1)^A,b^
		W	1.4 (0.3)^A,b^	1.5 (0.4)^A,b^	0.4 (0.1)^A,b^
		A	1.6 (0.5)^A,ab^	2.0 (0.4)^A,b^	0.6 (0.1)^A,b^
	crea.lign	C	0.8 (0.3)^A,b^	1.1 (0.1)^A,b^	0.5 (0.2)^A,b^
		W	1.5 (0.5)^A,b^	1.9 (0.8)^A,b^	0.6 (0.3)^A,b^
		A	1.2 (0.1)^A,b^	1.0 (0.1)^A,b^	0.6 (0.2)^A,b^
	ProBase	C	1.2 (0.1)^A,b^	1.9 (0.2)^A,b^	0.8 (0.3)^A,b^
		W	2.0 (1.0)^A,ab^	2.1 (1.1)^A,b^	0.7 (0.3)^A,b^
		A	0.5 (0.4)^A,b^	0.9 (0.5)^A,b^	0.6 (0.2)^A,b^
	Nexco	C	0.9 (0.2)^A,b^	1.3 (0.2)^A,b^	0.4 (0.1)^A,b^
		W	1.9 (0.4)^AB,ab^	2.3 (0.2)^A,b^	0.6 (0.2)^B,b^
		A	1.2 (0.1)^A,b^	0.9 (0.2)^A,b^	0.5 (0.1)^A,b^

Means that do not share a capital letter in the row and a small case letter in column are significantly different according to Tukey’s test (*p* < 0.05). *C*, coffee; *W*, wine; *A*, artificial aging

For ΔTP_00_, significance of factors (material, staining/aging, and interval) and interactions (material vs. staining/aging and staining/aging vs. interval) were detected (*p* < 0.001), as presented in Table 3. Staining in wine and coffee produced statistically similar or higher ΔTP_00_ than artificial aging in all tested materials and time intervals. ΔTP_00_ values were statistically greater after staining in wine than after artificial aging for the first (T0–T1) and overall time interval (T0–T2). Coffee produced statistically similar ΔTP_00_ as artificial aging in all intervals (*p* > 0.05).

**Table 3:** Means (SD) for staining- and aging-dependent changes in ΔTP_00_ for material vs. staining/aging interaction and staining/aging vs. time interval interaction (staining: T0—baseline, T1 and T2—60-h and 120-h exposure, respectively, and artificial aging T0—baseline, T1 and T2—150 kJ/m^2^ (T1) and 300 kJ/m^2^ (T2), respectively)

Material	Coffee	Wine	Aging
Beautifil	0.6 (0.6)^A,a^	0.4 (0.1)^AB,bc^	0.0 (0.1)^B,ab^
IvoBase	0.2 (0.5)^B,a^	1.0 (0.8)^A,a^	0.4 (0.4)^B,a^
crea.lign	0.2 (0.2)^A,a^	0.1 (0.3)^A,c^	0.0 (0.0)^A,ab^
ProBase	0.3 (0.3)^A,a^	0.5 (0.6)^A,b^	0.1 (0.4)^A,ab^
Nexco	0.2 (0.1)^AB,a^	0.6 (0.4)^A,ab^	− 0.1 (0.0)^B,b^
Staining/aging	T0–T1	T0–T2	T1–T2
Coffee	0.4 (0.4)^AB,b^	0.5 (0.4)^A,ab^	0.1 (0.1)^B,a^
Wine	0.7 (0.5)^A,a^	0.8 (0.6)^A,a^	0.1 (0.4)^B,a^
Aging	0.2 (0.3)^A,b^	0.2 (0.4)^A,b^	0.0 (0.3)^A,a^

Means that do not share a capital letter in the row and a small case letter in column are significantly different according to Tukey’s test (*p* < 0.05)

Results for ΔTP_ab_ show that differences in wine (ΔTP_ab_ range 0.4–2.0) were generally higher than those in coffee (ΔTP_ab_ range 0.2–1.8) and artificial aging (ΔTP_ab_ range 0.0–1.1). An exception is higher ΔTP_ab_ of Beautifil after staining in coffee, mean (SD) ΔTP_ab_ = 1.8 (0.6), than in wine, ΔTP_ab_ = 1.3 (0.3). ΔTP_ab_ values during the second time interval (T1–T2) were generally lower (ΔTP_ab_ range 0.0–0.5) albeit mostly without statistically significant difference than T0–T1 (ΔTP_ab_ range 0.0–1.8) and T0–T2 (ΔTP_ab_ range 0.0–2.0). The *R*^2^ values for the three-way ANOVA for ΔTP_00_ and ΔTP_ab_ were 55.6% and 66.3%, respectively.

As far as CIELAB color coorlinates are concerned, lightness of all materials decreased upon staining/aging (ΔL* range 0.1–1.9) except in PMMA-based ProBase and Ivobase CAD which exhibited higher L* after artificial aging (ΔL* range 0.8–0.9). Coordinate a* decreased in all materials (Δa* range 0.1–3.6), PMMA-based ProBase and Ivobase CAD exhibiting the smallest changes. Coordinate b* showed greatest variability depending on the material or staining/aging method, consistently increasing only in Beautifil and Crea.lign (Δb* range 0.3–4.4). Beautifil exhibited greater a* and b* coordinate changes as compared to other materials.

Gloss at baseline ranged from 76.7 to 87.0 GU. Beautifil exhibited the highest GU values, followed by Nexco, crea.lign, and IvoBase CAD, which showed similar GU values. Following staining and artificial aging, the lowest values of GR were recorded for Beautifil submitted to staining in wine for T0–T1 and T0–T2 intervals, followed by staining in coffee (Table 4). For gloss retention values, the *R*^2^ for the three-way ANOVA was 96.7%.

**Table 4:** Means (SD) for staining- and aging-dependent changes in gloss (GU) and gloss retention GR (%) of evaluated materials and time intervals (staining: T0—baseline, T1 and T2—60-h and 120-h exposure, respectively, and artificial aging T0—baseline, T1 and T2—150 kJ/m^2^ (T1) and 300 kJ/m^2^ (T2), respectively)

Material	GU	Staining/aging	GR (%)		
			T0–T1	T0–T2	T1–T2
Beautifil	87.0 (2.1) a	C	59.4 (2.2)^B,c^	62.5 (2.0)^B,c^	105.2 (1.4)^A,ab^
		W	50.8 (0.6)^B,d^	53.4 (1.2)^B,d^	105.4 (2.3)^A,a^
		A	98.0 (1.1)^A,ab^	96.1 (1.4)^A,ab^	98.1 (0.9)^A,c^
IvoBase	79.4 (2.1) b	C	97.6 (1.1)^A,ab^	99.8 (1.5)^A,a^	102.2 (1.0)^A, abc^
		W	94.5 (2.3)^A,b^	93.1 (2.0)^A,b^	98.6 (0.3)^A,c^
		A	98.4 (0.8)^A,ab^	95.8 (0.7^)A,ab^	97.3 (0.4)^A,c^
crea.lign	81.1 (2.5) b	C	99.4 (2.3)^A,ab^	100.2 (1.7)^A,a^	100.8 (1.3)^A,abc^
		W	101.5 (1.4)^A,a^	100.5 (1.8)^A,a^	99.0 (1.6)^A,bc^
		A	97.2 (1.9)^A,ab^	95.8 (3.1)^A,ab^	98.6 (1.7)^A,c^
ProBase	76.7 (3.4) c	C	96.0 (5.6)^A,ab^	97.1 (6.9)^A,ab^	101.1 (1.4)^A,abc^
		W	97.2 (5.5)^A,ab^	95.5 (5.9)^A,ab^	98.2 (1.7)^A,c^
		A	97.2 (4.1)^A,ab^	97.8 (4.7)^A,ab^	100.6 (2.2)^A,abc^
Nexco	81.5 (1.2) b	C	100.8 (0.8)^A,ab^	100.6 (1.3)^A,a^	99.8 (0.8)^A,abc^
		W	99.0 (1.9)^A,ab^	97.8 (1.3)^A,ab^	98.9 (1.1)^A,bc^
		A	97.3 (1.6)^A,ab^	94.8 (2.1)^A,ab^	97.4 (0.9)^A,c^

Means that do not share a capital letter in the row and a small case letter in column are significantly different according to Tukey’s test (*p* < 0.05). *C*, coffee; *W*, wine; *A*, artificial aging

**Fig. 1:**
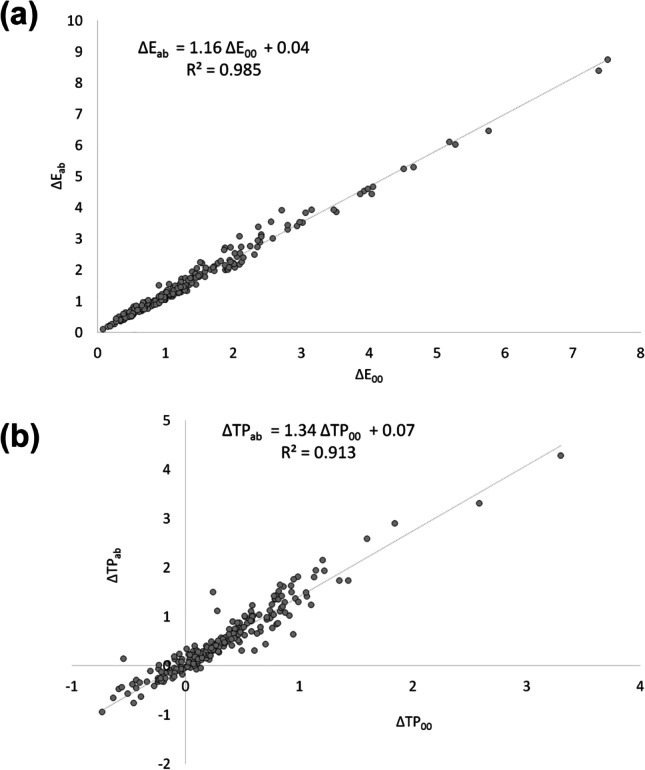
Pearson’s correlation between (a) Δ*E*_00_ and Δ*E*_ab_ and (b) ΔTP_00_ and ΔTP_ab_ values, coefficient of determination (*R*^2^) and regression equations for Δ*E*_ab_ and ΔTP_ab_ from the known Δ*E*_00_ and ΔTP_00_ values, respectively

Correlations between CIEDE2000 and CIELAB differences in color and translucency parameter are presented in Fig. 1. Pearson’s correlation has shown significant positive correlations between Δ*E*_00_ and Δ*E*_ab_, as well as between ΔTP_00_ and ΔTP_ab_ (*p* < 0.0001). The corresponding CIELAB to CIEDE2000 conversion equations are as follows: Δ*E*_00_ = 0.85*Δ*E_ab_ − 0.01 and ΔTP_00_ = 0.68ΔTP_00_ − 0.02.

## Discussion

Both research hypotheses were upheld as statistically significant color, TP, and gloss (GU and GR) differences were found between gingiva-colored resin-based restorative materials and after staining or artificial aging procedures as well as significant positive correlation between Δ*E*_00_ and Δ*E*_ab_ as well as between ΔTP_00_ and ΔTP_ab_.

Regarding the first research hypothesis, greatest Δ*E*_00 and_ Δ*E*_ab_ values were observed for Beautifil after staining in coffee and wine compared to other tested materials. As a giomer containing surface pre-reacted glass particles embedded in organic resin matrix, Beautifil is intended to combine good esthetic properties of resin-based composites and fluoride release and recharge potential from glass ionomers. Previous research showed greater water sorption by Beautifil compared to other tooth-colored materials [21]. Increased sorption and pigment uptake could be associated with greater color differences of gingiva-colored version of this giomer after staining in wine and coffee. This is supported by the absence of such color differences in Beautifil after exposure to xenon lamp weathering and lightfastness test chamber. Color differences of Beautifil were more strongly affected by changes in chromatic coordinates than the change in lightness. Another study also reported greater color differences in gingiva-colored Beautifil compared to gingiva-colored hybrid control composites [23]. The present results expand the range of gingiva-colored restoratives to PMMA-based and dimethacrylate-based materials showing better color stability than Beautifil giomer.

Light-cured, oligomer-based crea.lign and dimethacrylate-based Nexco showed similar Δ*E*_00_/Δ*E*_ab_ differences. crea.lign and Nexco contain different types and amounts of filler content albeit both have nano-sized filler particles. No data was found in the literature for gingiva-colored crea.lign or Nexco composite. Tooth-colored crea.lign and Nexco showed similar behavior in different experimental setups in that both exhibited greater color differences after exposure to curry than wine [18, 25]. Despite compositional differences, it appears that gingiva-colored versions of these materials undergo consistent color differences across a different spectrum of staining/aging procedures.

Wine induced the greatest Δ*E*_00_/Δ*E*_ab_ in all tested gingiva-colored-shaded materials, except IvoBase CAD, followed by coffee and artificial aging. Wine is known to one of the most potent discolorants of tooth-colored materials [17, 22] which is the reason for its frequent use to screen color stability of restorative materials. Previous studies also reported comparable or higher color differences induced by red wine than coffee in gingiva-colored resin-based restorative materials [7, 8, 24, 26] with curry being another potent discolorant for this type of materials [19]. Staining potential of wine is related to the pigments anthocyanins and tannins while acidity contributes to polymer matrix surface roughness and internal softening, and increased pigment sorption and adsorption, leading to extrinsic and intrinsic staining.

An exception in the present findings was IvoBase CAD whose Δ*E*_00_/Δ*E*_ab,_ values were affected by accelerated artificial aging to a greater extent than other materials. IvoBase CAD showed different changes in L*a*b* coordinates compared to other materials—increased L* and considerably decreased b* meaning aging resulted in material being lighter and less yellowish. Despite showing higher color differences after artificial aging, the results for gingiva-colored IvoBase CAD blocks were still lower compared to tooth-colored resin-based CAD blocks or laminate veneer materials in recent studies [28, 29] receiving the same energy as T1 in the present study. Industrially prepared PMMA-based CAD/CAM blocks appeared more resistant to structural, internal alteration and optical changes were rather associated with superficial effects of the staining/aging procedure. A recent study reported a similar effect of artificial aging on IPS Emax CAD, lithium disilicate glass ceramic, but not on zirconia reinforced lithium silicate and dimethacrylate-based CAD/CAM blocks which showed greater color differences after staining in wine/coffee [16]. Moreover, the same study showed that coffee had a more adverse effect on CAD/CAM restorative materials than wine or artificial aging [16]. These findings warrant further research into optical properties of gingiva-colored restoratives to discern critical factors that might adversely affect their clinical performance relative to various conditions in the oral environment.

In the present study ΔTP_00_ and ΔTP_ab_ of gingiva-colored restoratives were more affected by staining in wine than coffee or artificial aging. This is true in relation not only to the mean ΔTP_00_ and ΔTP_ab_ values, but also to the greatest range of ΔTP_00_ and ΔTP_ab_ differences between tested materials, from 0.1 in oligomer-based crea.lign to 1.0 in IvoBase CAD. Similarly greater effect of wine on translucency was reported for tooth-colored sculptable hybrid composites, which effect could be associated with greater sorption [17]. Greater ΔTP_00_ and ΔTP_ab_ in wine could be associated with reduced light transmission due to adsorbed and absorbed pigments, as well as structural polymer alterations caused by hydrolytic degradation [33], hygroscopic expansion, and polymer swelling [34]. Translucency differences in industrial PMMA-based CAD/CAM material IvoBase CAD are likely related to superficial pigment absorption and increased lightness.

As for gloss, giomer Beautifil showed the highest and PMMA-based ProBase the lowest baseline GU. The highest GU of Beautifil could be related to the filler particle type and polishability. Conversely, mixing could explain the lowest GU values for ProBase due to operator influence on powder-liquid dosage and quality of mixing. Despite having the highest GU at baseline, Beautifil showed the lowest GR in colored beverages. A recent study tested a gingiva-colored giomer, similar to the one used in the present study, and reported that thermal and acidic conditions significantly affected its surface properties [23]. The present findings confirm that gloss of gingiva-colored Beautifil giomer is also less resistant to acidic and alcoholic challenge. This may be explained by softening the organic matrix and increased surface roughness as was reported for tooth-colored Beautifil giomer in a previous study [19]. Gloss of other tested materials was little affected by staining or accelerated artificial aging as evidence by more than 90% GR after these procedures.

Regarding the second research hypothesis, significant positive correlation between Δ*E*_00_/Δ*E*_ab_ and ΔTP_00_/ΔTP_ab_ has been established in the present study. Similar correlation between Δ*E*_00_/Δ*E*_ab_ was reported for denture resins after staining/cleansing cycles [9]. Regression equations were presented to allow the results of studies using only one of these formulas to be converted and compared with the present study. It is important to highlight that the reported equations are valid for the methodology used in the present study.

**Fig. 2:**
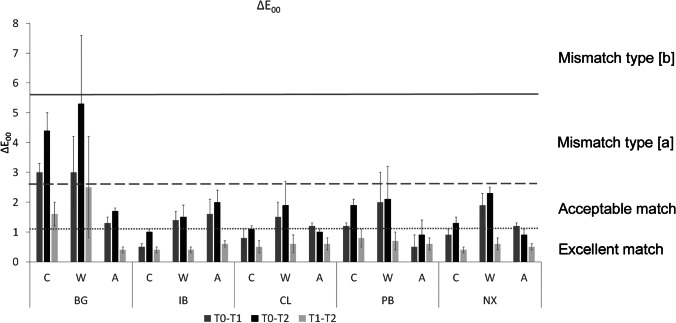
CIEDE2000 color differences (Δ*E*_00_) compared with literature data for visual thresholds for gingiva and gingiva-colored materials [15]. Excellent match: Δ*E*_00_ ≤ 1.1 or Δ*E*_ab_ ≤ 1.7 (PT); acceptable match: Δ*E*_00_ > 1.1 and 2.8 or Δ*E*_ab_ > 1.7 and ≤ 3.7 (AT); mismatch type [a]: Δ*E*_00_ > 2.8 and ≤ 5.6 or Δ*E*_ab_ > 3.7 and ≤ 7.4 (> AT and ≤ AT × 2); mismatch type [b]: Δ*E*_00_ > 5.6 and ≤ 8.4 or Δ*E*_ab_ > 7.4 and ≤ 11.1 (> AT × 2 and ≤ AT × 3); and mismatch type [c]: Δ*E*_00_ > 8.4 or Δ*E*_ab_ > 11.1 (> AT × 3). C, coffee; W, wine; A, artificial aging; BG, Beautifil; IB, IvoBase; CL, crea.lign; PB, ProBase; NX, Nexco Paste

Color differences observed in the present study were interpreted based on literature data on CIEDE2000 visual thresholds (Fig. 2) [15]. Excellent match was found for crea.lign after artificial aging and staining in coffee, IvoBase CAD after staining in coffee, and ProBase and Nexco after artificial aging (Δ*E*_00_ ≤ 1.1) at the final observation period (T0–T2). Δ*E*_00_ of crea.lign and Nexco after artificial aging at T0–T1 was just into the “acceptable match” range while it was considered “excellent match” at T0–T2. Table 2 shows that mean (SD) of Δ*E*_00_ of CL and NX at T0–T1 and T0–T2 was very close (1.2 (0.1) vs. 1.0 (0.1) and 1.2 (0.1) vs. 0.9 (0.2), respectively) with no statistically significant differences. Clinical meaning of this result is that color differences of CL and NX associated with artificial aging occur during the initial observation period and reach the threshold between the “excellent” and “acceptable” match.

Beautifil showed *Δ*E_00_ (Δ*E*_00_ range 1.7–5.3) which corresponded to acceptable match after artificial aging and mismatch [a] after staining. After wine staining, all materials except Beautifil exhibited acceptable match (Δ*E*_00_ range 1.5–2.3), with additionally acceptable match observed for ProBase and Nexco after coffee staining (Δ*E*_00_ range 1.3–1.9). As for Δ*E*_ab_ related to CIELAB visual thresholds, excellent match was recorded for IvoBase CAD, crea.lign, and Nexco after coffee staining, as well as crea.lign, Nexco, and ProBase after artificial aging (Δ*E*_ab_ range 1.0–1.4). Acceptable match was recorded after wine staining in all materials except Beautifil (Δ*E*_ab_ range 1.8–2.9), after coffee staining in ProBase and after artificial aging in IvoBase CAD (Δ*E*_ab_ range 2.0–2.9). The only material showing mismatch [a] related to CIELAB thresholds was Beautifil after coffee and wine staining (Δ*E*_ab_ range 5.0–6.1).

Time-dependent changes in color and translucency differences were observed in the present study. Greater color and translucency differences occurred during the first (T0–T1) than the second observation period (T1–T2) indicating a non-linear effect of staining/artificial aging on this material property. Previously, time-dependent mass and volume changes in tooth-colored resin-based composites were found to be non-linear with steep inclines initially, following a slower rise or a plateau effect over the remaining period of immersion [35]. Similar time-dependent color and translucency differences were found for gingiva-colored restorative materials when exposed to staining or accelerated artificial aging.

Five gingiva-colored, resin-based restorative materials with significant compositional differences were tested in this study. These differences included different organic resin matrices (dimethacrylate, oligomer, or polymethyl-methacrylate), inorganic filler types (nano-ceramic filler, S-PRG filler, or silicon dioxide filler particles), and polymerization types (light-cured, heat-cured, pre-prepared CAD/CAM block). This approach allowed a broad insight into a range of different resin-based materials with the same indication, gingiva replacement.

The main limitation of this study is that it was in vitro simulation which does not allow for replicating complex interplay of factors in vivo. The results may not be directly translated into clinical setting. However, this and similar laboratory setups allow material comparison under the same experimental conditions and serve as good starting point for initial material comparison. One could argue that a limitation is the lack of “distilled water” group as a control. Artificial aging is defined by the ISO to simulate material aging in the mouth under normal conditions when not exposed to colored beverages. Water aging only is an oversimplification of the actual clinical situation. Adding water to this study would have increased the total number of groups by 15 (5 materials and 3 intervals) requiring significantly more specimens to maintain the power of the study. Information obtained by comparing color, translucency, and gloss differences between water aging alone with artificial aging or wine/coffee staining is considered not clinically relevant to justify adding this aging method.

Despite different finishing and polishing toolkits recommended by manufacturers for their respective materials, the same multi-step polishing protocol was adopted for all tested materials for two reasons: (1) finishing and polishing procedures in manufacturers’ Instructions for Use (IFU) for each material are not sufficiently detailed to be reproduced and (2) there is no information in the IFU files pertaining to surface roughness of each material using the manufacturer’s recommended finishing and polishing toolkit suggesting that initial surface roughness of the tested materials would be the same. Besides, in the present study, we measured the relative change in surface roughness (gloss retention, %) of the tested materials before and after staining/aging.

While there may be differences in the tested properties depending on the storage prior to staining/aging, there is no standard for storage conditions of gingiva-colored materials prior to testing. Measurements were done after polishing and 10 min of ultrasonic cleansing in deionized water. It was decided to not store specimens for 24 h in distilled water as this is an arbitrary period generally adopted in material testing, but which does not reflect clinical conditions. It is more important that all specimens were subjected to the same conditions. In clinical conditions, materials are exposed to artificial aging immediately after placement while exposure to staining solutions is variable.

The question of the relationship between the artificial aging setup and years of clinical service has not been answered in the literature. The amount of exposure to ISO 4892–2 required to simulate certain clinical service time depends on oral hygiene, dietary habits, bleaching, smoking, and other patient-related factors, but also on the tested material as sensitivity to the degradation factors produced by the accelerated test is different among materials. Even if one would calculate a specific user/material correlation between, e.g., 150 or 300 kJ/m^2^ and months/years in service, it would not be universally applicable. Moreover, the manufacturer’s estimation that 300 h of aging is approximately equivalent to 1 year of clinical service may not be accurate [36, 37]. The variables mentioned above and differences among weathering devices and aging cycles influence the results and consequently the accuracy of estimation. When designing artificial aging experimental setup, standardizing to a specific amount of radiant exposure is more important than the actual clinical service time and provides the same testing condition to all evaluated materials. In addition, the total exposure amounts are such that they should not (a) fail materials that would be suitable for this application, or (b) pass materials that may not be suitable for this application.

## Conclusions

Based on the present findings, it was concluded that:

1. Color, translucency, and gloss changes of gingiva-colored resin-based restorative materials were material- and staining/aging-dependent and generally increased with exposure.

- Giomer Beautifil showed the most pronounced color differences, while color differences of IvoBase CAD were higher after exposure to accelerated artificial aging than upon staining in colored beverages. Wine induced the greatest color differences followed by coffee and artificial aging in all materials except IvoBase CAD.
- Translucency parameter changes were the smallest in oligomer-based crea.lign and the greatest in PMMA-based IvoBase CAD. Wine induced the greatest differences in translucency parameter, followed by coffee and artificial aging.
- Although the initial gloss was material-dependent, gloss retention was above 90% for all materials except giomer Beautifil.

2. Significant positive correlation was found between Δ*E*_00_/Δ*E*_ab_ and ΔTP_00_/ΔTP_ab_.
